# Strange features are no better than no features: predator recognition by untrained birds

**DOI:** 10.1007/s10071-024-01924-z

**Published:** 2025-01-07

**Authors:** Ondřej Fišer, Irena Strnadová, Petr Veselý, Michaela Syrová, Michal Němec, Barbora Kamišová, Josef Šalom, Roman Fuchs

**Affiliations:** 1 Department of Zoology, Faculty of Science, University of South Bohemia, Branišovská 1760, České Budějovice, 370 05 Czech Republic; 2 Department of Zoology, Faculty of Science, Charles University, Albertov 6, Prague 2, 128 00 Czech Republic

**Keywords:** Antipredator behaviour, *Lanius collurio*, Predator–prey interactions, Recognition, Categorization, Mobbing

## Abstract

Predator recognition is essential for prey survival, allowing for appropriate antipredator strategies. Some bird species, such as the red-backed shrike (*Lanius collurio*), distinguish not only between predators and non-threatening species but also between different predator species. Earlier studies have identified general predator “key features”, especially beak shape and talons, as critical for predator recognition. The question, though, still remains of whether exchanging predator key features with those of nonpredatory species or, alternatively, completely removing them, have different or equal impact on recognition. Here we tested to ascertain whether the presence of the “incorrect key features” of a harmless pigeon (*Columba livia*) placed on a common kestrel (*Falco tinnunculus*) body impairs predator recognition more efficiently than the absence of any key features. We presented an unmodified kestrel dummy and two modified kestrel dummies (one with pigeon key features, the other lacking key features) to wild red-backed shrikes defending their nest. The shrikes attacked the unmodified dummy kestrel more intensively than both kestrel modifications when defending the nest. However, shrikes did not show different responses to the kestrel with pigeon key features and the featureless kestrel. Our findings show that the absence and exchange of key features have the same effect in this case. These results are discussed in the context of recognition of a specific predator species and predators as a category in general.

**Supplementary Information:**

The online version contains supplementary material available at 10.1007/s10071-024-01924-z.

## Introduction

Predator recognition is crucial for the survival of all animals (Lima and Dill 1990). The chances of survival are undoubtedly increased by the most accurate determination of imminent danger. It would be advantageous for the prey to estimate whether the predator is dangerous, even if its specific species is unfamiliar (Ferrari et al. 2016; Carthey and Blumstein 2018; Ehlman et al. 2019). Birds are able to distinguish not only harmless heterospecifics from predators (Kullberg and Lind 2002; Salazar et al. 2023) but also individual predator species from each other (Templeton et al. 2005; Dutour et al. 2016). Even within individual predator species, a distinction can be made between adults and juveniles (Špička et al. 2024). The most accurate recognition of such differences is advantageous for potential prey, as prey can then choose an appropriate antipredator strategy towards them (Caro 2005; Blumstein 2006; Fuchs et al. 2019).

One of the key questions is how a predator is recognised. This phenomenon was first studied by classical ethologists in the last century. They worked with the term “key features” (Lorenz 1939; Krätzig 1940; Tinbergen 1948). These are features that alone allow birds to distinguish a predator from a harmless animal. They tested responses to flying bird dummies, and the key feature here was the shape of the bird’s silhouette differentiating an aerial predator from a goose. Other studies used dummies of perching birds and focused primarily on more detailed features that also enable birds to discriminate aerial predators from harmless birds, notably beak shape (Curio 1975; Gill et al. 1997; Beránková et al. 2014) and eye colour (Curio 1975; Scaife 1976; Beránková et al. 2014). However, some studies have shown that potential avian prey also discriminate between different species of aerial predators using both size (Klump and Curio 1983; Templeton et al. 2005; Palleroni et al. 2005; Courter and Ritchison 2010; Beránková et al. 2015; Antonová et al. 2021; Fišer et al. 2024) and colouration (Curio 1975; Beránková et al. 2015; Veselý et al. 2016; Antonová et al. 2021). Moreover, the ability to recognise a predator is also affected by experience (Carlson et al. 2017). The question arises as to the nature of the relationship between features characterising all aerial predators and features characterising individual species of birds of prey or owls.

Němec et al. (2021) manipulated the general avian-predator key features (curved beak, talons, supraorbital ridges, hereafter referred to as AP key features) and species-specific colouration of the common kestrel (*Falco tinnunculus*), a predator of juvenile red-backed shrikes (*Lanius collurio*). They found that the shrike does not perceive the modified kestrel as a dangerous predator. Namely, if it has retained the colouration but the key features of a bird predator have been replaced by those of a harmless pigeon (*Columba livia*). On the other hand, a kestrel with preserved AP key features, but with colouration interchanged with another avian predator unknown to the shrikes, the black baza (*Aviceda leuphotes*), was not recognised as a dangerous predator either. Thus, the results demonstrate that the manipulation of key features crucially influenced the ability of shrikes to recognise predators. However, it is not entirely clear what causes this influence. Shrikes could either have been responding to the absence of AP key features or to the presence of key features of a harmless bird species, the pigeon. Testing for these possible responses was the aim of this paper.

We used the same study species (red-backed shrike) and context (nest defence) as the study done by Němec et al. (2021), but we chose to alter the predators differently. We added one new modification and used one modification from a previous study, namely either with the key predator features exchanged with those of a harmless pigeon or with the key features (beak, eyes, talons) completely removed and with only body position, shape, size and colouration preserved.

We hypothesised that predator recognition would worsen in both modifications as neither had the AP key features. Nevertheless, one had the key features of a harmless bird, while the other had no key features. If the shrikes recognised the key features of harmless birds, the ability to recognise a kestrel in this dummy would be lower than in the case of a dummy without any key features.

We predicted that:

1. Shrikes would attack the two modified dummies less than the unmodified kestrel.
2. Shrikes would attack the dummy with pigeon key features less than the dummy with no key features.

## Methods

### Study area and material

Behavioural experiments were conducted during two breeding seasons (June–July) in 2012–2013 in the Doupov mountains, Western Bohemia, Czech Republic (GPS: 50.17 N, 13.14E). The experiment was carried out in the vicinity of nests containing nestlings ranging in age from 6 to 14 days. We therefore expected parents to be highly motivated to defend them (Strnadová et al. 2018). The nests at this site are distributed across meadows and pastures mainly in thorny shrubs. A total of 18 active nests were tested and included in the analyses (*N* = 18).

## Study species and experimental stimulus

The red-backed shrike (*Lanius collurio*) is known for the aggressiveness with which both parents defend their nest (Tryjanowski and Goławski 2004). The ability of the species to recognise predators and respond appropriately to them is what has been extensively studied thus far. Shrikes distinguish between predators according to their level of perceived danger and their key characteristics (Němec et al. 2021). Its antipredator behaviour is then adapted according to the risk of self-injury or the likelihood of survival of nestlings or eggs in the nest caused by the attacking predator (Strnadová et al. 2018).

Our experiment took place during the nesting period, so we chose the common kestrel (*Falco tinnunculus*) as a predator, which, apart from small rodents, is also able to hunt passerine nestlings (Riegert and Fuchs 2011). The shrikes at the study site are very familiar with kestrels and actively chase them away from their nests (Strnad et al. 2012). Thanks to its specific colouring, the common kestrel cannot be mistaken for any other familiar predator. At the same time, it bears the typical key features of a predator (curved beak with yellow cere, yellow talons, supraorbital ridges, and yellow ring of bare skin around brown eyes).

**Fig. 1:**
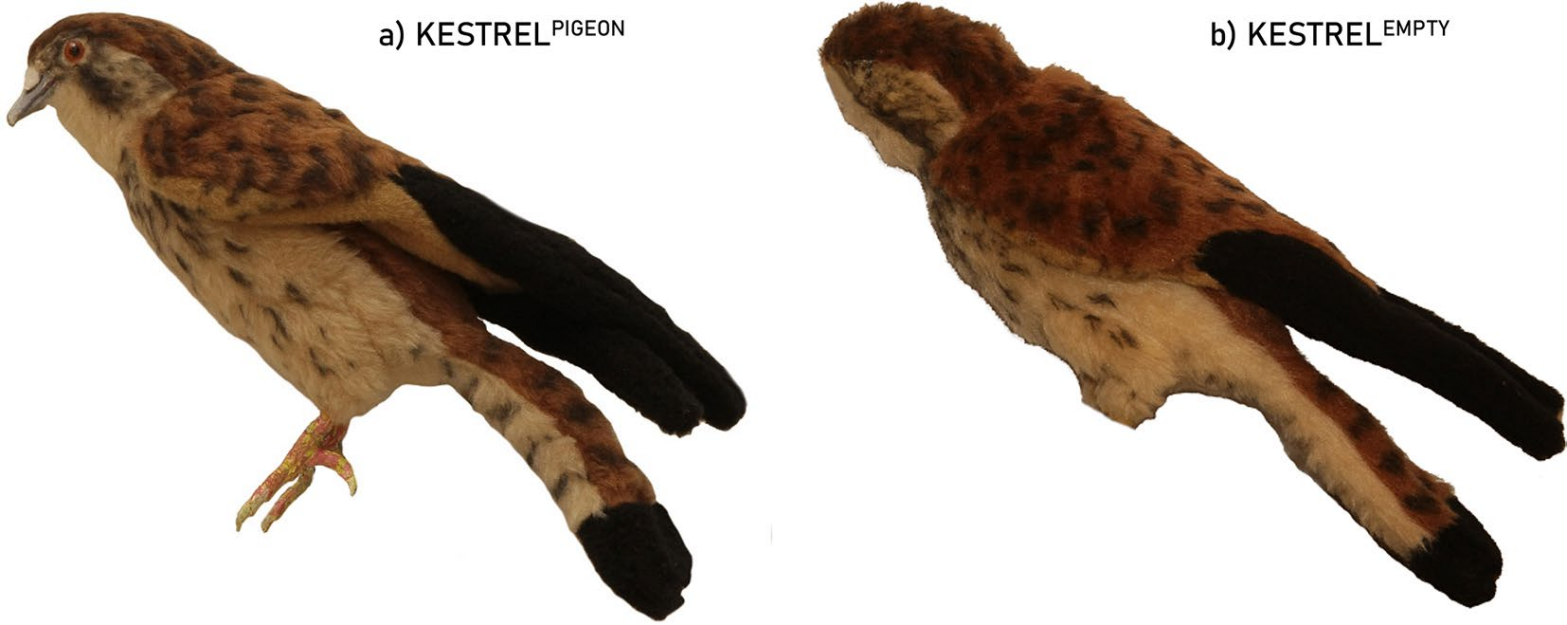
Photographs of modified dummy kestrels presented in the proximity of the red-backed shrike nest: (**a**) kestrel with pigeon key features; (**b**) kestrel with missing key features

The kestrel was presented in two life-size body modifications (Fig. 1). One modification consisted of replacing AP key features with pigeon key features (straight beak with white cere, pink legs with straight short claws, orange-red eyes without supraorbital ridges and bare skin) here referenced as KESTREL^PIGEON^ (Fig. 1a). The second modification consisted of a complete absence of key features (eyes, beak, talons) = KESTREL^EMPTY^ (Fig. 1b). The gaps left by the absence of the key features were filled in with the same feather-like surface, and were of the same colour, as the surrounding body surface. Both modifications retained the colouration typical of a female kestrel. The dummies were prepared from textiles and stuffed with cotton with a skull carrying the beak and legs made of modelling clay. The eyes were made of glass and their colour and size were adjusted to particular bird species. The dummy was painted with acrylics, the surface of the hairy textile with a paint layer imitating feathers quite well. The effective use of such artificial dummies has been demonstrated several times in previous experimental studies involving shrikes (Němec et al. 2021; Krausová et al. 2022).

In addition to these two dummies, we used an unmodified stuffed kestrel (KESTREL) and a stuffed domestic pigeon (*Columba livia*) (PIGEON) as baseline stimuli.

## Experimental design

Once an active nest was found, checks to assess the breeding stage were made under adequate climatic conditions (dry, sunny) to avoid endangering the nest. If the nestlings had reached at least 6 days of age, the experiment took place; otherwise, the parents might have abandoned the nest. The experiment took place during the day, again, under adequate climatic conditions, and always between 9:00 and 19:00. Each dummy was presented approximately 1 m from the nest on a 1.5 m high pole so that the predator was in the position of perching on a branch (wings folded and head facing the nest). All four dummies (baseline kestrel, baseline pigeon, and two kestrel modifications) were presented at one nest in a balanced randomised order. Each set of trials was conducted during a single day to minimise stress and the effect of nestling age on the behaviour of the parents. There was an hour interval between each dummy trial, which served to allow the parents to calm down and also to provide the nestlings with food. The experiment itself lasted for 20 min from the time the dummies were noticed by the parents. After 20 min, if no shrike appeared, the dummy trial was terminated and considered as a zero value for all recorded behaviours. The observer was approximately 50 m from the nest and observed the nest with binoculars and recorded the bird’s behaviour with commentary on a video camera.

## Recorded behaviour and statistical analysis

We used the total number of attacks on the dummy as the sole behavioural proxy of nest defence and aggression for each shrike parent in the defending pair in our analyses (Carlson and Griesser 2022). Attacks were recorded whenever a bird flew towards the dummy and lowered its flight height just before the dummy. The attacks may or may not have been associated with physical contact with the dummy. We also performed exploratory visualization of the behavioural response of individual nests to individual dummies to describe behavioural variability.

The number of attacks was log-transformed (log10(number_of_attacks + 0.5)) to meet the requirements for a normal distribution. Each pair was tested with four dummies and the attacks of each parent were recorded, so we chose to explain the variation in attacks using linear mixed-effects models (LMM, command lmer in the lme4 package). Nest identity was entered into the model as a random factor, and the identity of each bird was nested within the nest.

We explained the total number of attacks using the following predictors: the dummy and the order in which each was presented. Factors were entered sequentially into the model using “stepwise forward selection” and the models were compared using the likelihood-ratio test (χ^2^ test). The Tukey HSD post-hoc test (command glht in the multcomp package) with the Tukey correction for repeated measures was used for pairwise comparisons of levels of significant categorical predictors. All analyses were performed in the R Environment (R Core Team 2020).

## Ethical note

Permission for studies on wild red-backed shrikes was granted by the Ministry of the Environment of the Czech Republic (13842/2011-30), the licences permitting experimentation with animals no. CZ02759 and CZ02766 were offered by the Ministry of Agriculture of the Czech Republic. This research adhered to the ASAB/ABS guidelines for the use of animals in research. The authors declare that the experiments comply with the current laws of the Czech Republic and the European Union. The shrikes were tested in their natural environment with minimum disturbance and experienced natural levels of stress.

## Results

Only the dummy type significantly affected the number of attacks in the experiments. The effect of the sequence of the trials was not significant (Table 1).

**Table 1:** Effects of predictor variables in particular models (LMM, Likelihood ratio test, see methods section for further details)

Response	Predictor	AIC	BIC	χ2	DF	*P*
attacks	dummy	**259.73**	**280.52**	**22.73**	**3**	**< 0.01**
	sequence	264	293.70	1.73	3	0.63

Significant effects are in bold. AIC - Akaike’s Information Criteria, BIC - Bayesian Information Criteria, χ2 – Chi-square, DF – Degrees of freedom

**Fig. 2:**
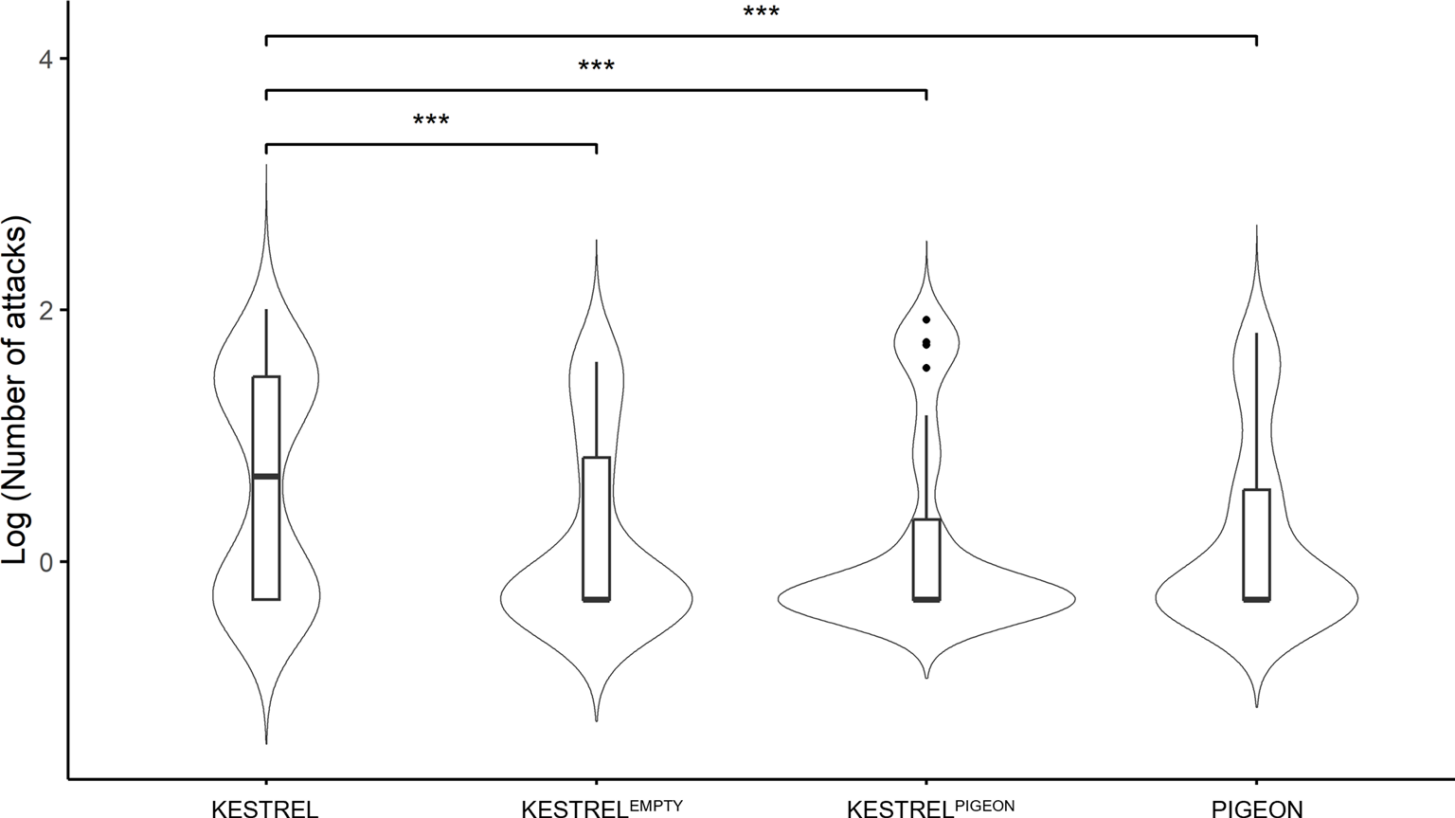
The logarithmic number of attacks performed by individual shrikes towards the dummies in the experiment. Individual symbols *** indicate significance levels *P* < 0.001. The sample size is 18 breeding pairs. The thick line within each box represents the median, the vertical span of the box represents the range from the lower (25%) to the upper quartile (75%), the so-called IQR – interquartile range; and the range of whiskers represents the nonoutlier minimum and the maximum. Black dots represent outliers above the upper quartile + 1.5 * IQR. The violin shape displays a kernel density estimation, illustrating the distribution of the data. Wider sections of the violin plot indicate higher probabilities of observing the corresponding values in the data, while narrower sections reflect lower probabilities

Post hoc comparisons showed that the pigeon dummy (PIGEON) was attacked significantly less often than the baseline kestrel dummy (KESTREL) (Tukey HSD: *Z* = 3.98, *P* < 0.01). However, there was no difference between PIGEON and the kestrel dummy with pigeon features (KESTREL^PIGEON^) and PIGEON and the kestrel dummy without any features (KESTREL^EMPTY^) (Tukey HSD: *Z* values always below 1 and *P* values above 0.99, Fig. 2). Moreover, the baseline KESTREL dummy was attacked significantly more often than both modified variants KESTREL^EMPTY^ and KESTREL^PIGEON^ (Tukey HSD: *Z* values always above 4 and *P* values below 0.01, Fig. 2). There was no significant difference between the KESTREL^PIGEON^ and KESTREL^EMPTY^ (Tukey HSD: *Z* value below 1, *P* value above 0.99, Fig. 2).

To show the variability between the behavioural responses of individual nests, we visualized the sum of attacks of each nest on each dummy and divided them into three groups with similar behavioural patterns. The graph shows that 7 pairs reacted to almost none of the dummies and 3 pairs reacted very intensely to all dummies compared to the unmodified kestrel. The rest of the nests responded to the unmodified kestrel more than to the control harmless pigeon as anticipated (Fig. 3 in Online resource 1 in Supplementary material).

## Discussion

Consistent with our first prediction, the red-backed shrikes attacked the unmodified dummy kestrel (KESTREL) more intensively than the two kestrel modifications when defending their nests. However, the shrikes did not attack the kestrel without eyes, beak, and talons (KESTREL^EMPTY^) more than the kestrel with pigeon eyes, beak, and claws (KESTREL^PIGEON^). Moreover, the shrikes did not attack either of the modified kestrels more than the pigeon (PIGEON). Thus, our hypothesis that the presence of the eyes, beak, and claws of a harmless pigeon reduces the ability of shrikes to recognise a dummy kestrel more than the absence of eyes, beak and talons was not confirmed. Only the presence or absence of the correct key features of the kestrel determines recognition. This finding is also supported by the results of Němec et al. (2021), who showed that the shrikes attacked the kestrel dummy only if it bore both the species-specific colouration and the raptor beak, eyes and talons.

Our data contain a rather large variation of attacks by individual nests. The variability in the behavioural responses of the defending parent pairs is characteristic of all studies on the defensive behaviour of red-backed shrikes (Strnadová et al. 2018). Active defence is demanding and some pairs are not capable of it in the final stage of breeding, when the nutrition of the young is most demanding (Montgomerie and Weatherhead 1988). Other pairs, on the other hand, attack every intruder in close proximity to the nest and do not assess differences in their danger, which may be due, for example, to individual differences in experience or personality (Vrublevska et al. 2015). However, the results show that the group of individuals that attacked in accordance with normal shrike nest-defence behaviour (an unmodified kestrel is attacked more than a harmless control pigeon) did not show any difference between the two modified kestrel dummies.

Nevertheless, what unites all three dummies is the characteristic body colouration but also the body size of the common kestrel. This colouration is fully preserved in all dummies, yet we show that it hardly secures proper recognition of the kestrel only by itself. However, Strnad et al. (2012) showed that the red-backed shrike is familiar with the colouration of the common kestrel and is able to use it for kestrel discrimination from another similarly sized aerial avian predator, the Eurasian sparrowhawk (*Accipiter nisus*). Yet neither a kestrel with unmodified colouration, but with the eyes, beak, and claws of a harmless pigeon, nor one without the eyes, beak and talons of a kestrel, is recognised as a kestrel. Two explanations are offered. The shrikes could recognise that these modified predators do not have the potential (absence of a hooked beak and long sharp claws) to cause physical harm to themselves or their young. The second possibility is that the shrikes did not recognise the kestrels in the dummies; in other words, they did not generalise their knowledge of known exemplars (sensu Huber and Aust 2006; Lea et al. 1993) or prototypes (sensu Aydin and Pearce 1994; Huber and Aust 2006) of the kestrel to the presented modified kestrel dummies.

It has been previously shown that birds’ predator recognition or behavioural response is impaired when a stimulus is modified. In the study of Beránková et al. (2014) great tits (*Parus major*) showed decreased levels of fear in response to the substitution of the beak, talons and eyes on the sparrowhawk dummy for the beak, claws, and eyes of the pigeon. On the contrary, the beak, talons, and eyes of the sparrowhawk placed on the pigeon’s dummy were not sufficient to elicit a fear response. According to the authors, these results suggest that AP key features alone are again not sufficient in eliciting an antipredator response. This is consistent with the findings of Němec et al. (2021) that the change in colouration of the predator with unmodified AP key features also reduced the antipredator behaviour of red-backed shrikes. These studies, similar to our results, show that the process of aerial predator recognition requires the presence of AP key features as well as species-specific colouration.

The importance of specific key features in stimuli recognition has been tested in studies using the discrimination learning paradigm (e. g. Huber and Aust 2006). Aust and Huber (2002) presented picture stimuli that were modified in different ways to pigeons trained to recognise human figures. The authors summarise that pigeons did not explicitly rely on a single feature (e.g. skin, legs, body position) to constitute a given category. At the same time, they do state that some features of a given category are more defining for pigeons than others. However, if there were more than one of the features present on a given stimulus, then appropriate categorization was more likely to occur. Compared to our results and those of Němec et al. (2021), pigeons were able to recognise the human figure even in substantially modified stimuli, while the nest-defending red-backed shrikes were not able to categorise the modified predator dummies. The reason for this discrepancy may reside in different motivations. The pigeons were trained to look for the human figure, whereas shrikes activate their nest defence behaviour only when the kestrel is appropriately recognised.

If we combine the results of our experiment with those of Němec et al. (2021), two possible explanations are offered. First, the shrikes may require the joint presence of AP key features and species-specific kestrel colouration to recognise the presented stimulus as a kestrel. Second, recognition of the kestrel may be a two-step process. The shrikes will first include the presented stimulus in the broader category of aerial predators given the presence of the AP key features; and second, will use appropriate colouration to place it in the narrower category of kestrel. Such a process would better explain why shrikes do not respond to overall colouration, even though it is a more conspicuous feature than beak or talon shape. A stimulus that does not fall into the aerial predator category needs no further attention.

## Conclusion

Our study confirms the importance of avian predator key features in predator recognition by untrained birds. Following the work of Němec et al. (2021), we show that red-backed shrikes rely on both species-specific colouration and AP key features (eyes, beak, and talons) to recognise the kestrel. While shrikes attacked unmodified dummy kestrels more intensely than modified versions, they did not discriminate between kestrels with pigeon-like features and those without any key features. Hence, the presence of pigeon-like features does not affect the recognition process, only the absence of AP key features is important. Taken together, shrikes may not recognise modified kestrels as a threat due to the absence of dangerous features and thus the detailed species-level recognition of the kestrel does not even start. These findings, along with previous research, suggest that predator recognition in red-backed shrikes is a complex process requiring the integration of multiple features. Future studies should further investigate the sequential recognition process and its neurophysiological basis to understand better the cognitive mechanisms that drive antipredator behaviour.

## Electronic supplementary material

Below is the link to the electronic supplementary material.

Supplementary Material 1

Supplementary Material 2

## Data Availability

Original data is provided within the supplementary information file.
