# Supplementary material for: Strange features are no better than no features: predator recognition by untrained birds
Source: Anim Cogn. 2025 Jan 7;28(1):5. doi: 10.1007/s10071-024-01924-z (PMC11706896; doi:10.1007/s10071-024-01924-z)
Supplement: Supplementary file 1 — Supplementary Material 1 [file 10071_2024_1924_MOESM1_ESM.pdf]

Strange features are no better than no features: predator recognition by untrained birds

Animal Cognition

Ondřej Fišer[a], Irena Strnadová[b], Petr Veselý[a],*, Michaela Syrová[a], Michal Němec[a], Barbora Kamišová[a], Josef Šalom[a], Roman Fuchs[a,b]

Affiliation: [a]Department of Zoology, Faculty of Science, University of South Bohemia, České Budějovice, Czech Republic, [b]Department of Zoology, Faculty of Science, Charles University, Prague, Czech Republic

Correspondence: Petr Veselý, Department of Zoology, Faculty of Science, University of South Bohemia, Branišovská 1760, 37005 České Budějovice, Czech Republic

E-mail address: veselp03@prf.jcu.cz

Telephone number: CZ +420 387 772 241

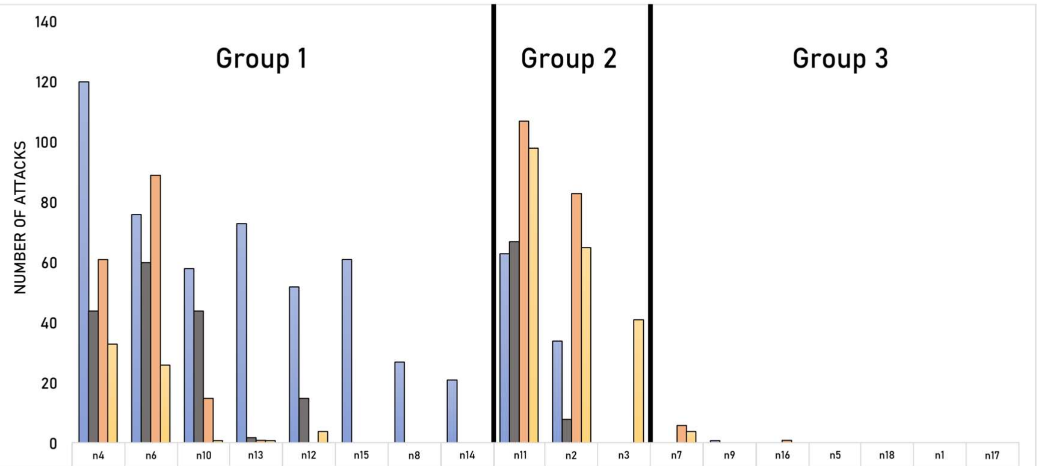

	n4	n6	n10	n13	n12	n15	n8	n14	n11	n2	n3	n7	n9	n16	n5	n18	n1	n17
KESTREL	120	76	58	73	52	61	27	21	63	34	0	0	1	0	0	0	0	0
KESTREL_EMPTY	44	60	44	2	15	0	0	0	67	8	0	0	0	0	0	0	0	0
KESTREL_PIGEON	61	89	15	1	0	0	0	0	107	83	0	6	0	1	0	0	0	0
PIGEON	33	26	1	1	4	0	0	0	98	65	41	4	0	0	0	0	0	0

Fig. 3 The graph shows the variation in the number of attacks by parental pair on each type of dummy within each nest. The nests are divided into three groups: Group 1) Nests that attacked the unmodified kestrel most compared to the harmless control pigeon; Group 2) Nests that attacked the harmless control pigeon and other dummies significantly more compared to the unmodified kestrel; Group 3) Nests that did not attack at all or attacked minimally any dummy.
